# Profiles of Innate Immune Cell Infiltration and Related Core Genes in Psoriasis

**DOI:** 10.1155/2021/6656622

**Published:** 2021-02-23

**Authors:** Xiaoting Gong, Wei Wang

**Affiliations:** Department of Clinical Laboratory, Tongde Hospital of Zhejiang Province, Hangzhou, Zhejiang 310012, China

## Abstract

Psoriasis is an inflammatory skin disease with substantial morbidity. Numerous patients with psoriasis experience recurrence after therapy. The underlying mechanism about psoriasis is still not fully understood. Some evidences suggest that innate immunity may play an unexpected and important role in active severe psoriasis. In this work, the deconvolution algorithm CIBERSORT was conducted to identify the infiltration of innate immune cells and related core genes in psoriatic plaque. Datasets from the Gene Expression Omnibus, including skin samples from 405 psoriasis patients and 91 healthy donors, were downloaded for analysis. Considerable differences of the innate immune cell composition were uncovered between psoriatic plaque and control skin. Results revealed that *γδ* T cells, resting NK cells, M0 macrophages, M1 macrophages, activated dendritic cells, and neutrophils were significantly increased in psoriatic skin, while resting mast cells and active NK cells were significantly decreased. Moreover, the proportion of M0 macrophages or resting mast cells was found to be associated with disease severity. Spearman correlation analysis suggests that RORC and S100A12 genes were related to disease severity, while genes including S100A12, CLEC4C, IL-19, AIM2, IL-17F, and PPARGC1A were correlated with biologic treatment response. In conclusion, this work displays innate immune status in psoriatic skin and provides novel clues for clinical decisions and mechanism study.

## 1. Introduction

Psoriasis is a chronic inflammatory skin disorder, which is related to multiple comorbidities and affects 125 million people worldwide [1]. It substantially diminishes life quality of patients and is incurable. It could be recurrent under stimulation with risk factors, such as skin trauma, infection, stress, and certain medications including interferon, *β* blocker, and lithium [2]. Psoriasis is thought to be triggered by innate and adaptive immune components, interacting with skin tissue cells [3]. Numerous studies point that interleukin- (IL-) 23/IL-17 axis plays a central role in psoriasis pathogenesis [3]. It has been validated by the therapeutic efficacy of blocking the IL-23 and IL-17 pathway in moderate-to-severe psoriasis, such as ustekinumab and brodalumab [4]. However, these clinical methods have limitations, and symptoms are likely to reappear upon cessation of therapy.

Accumulating circumstantial evidences suggest that adaptive immune response is more likely to play a critical role in mild stable psoriasis; in contrast, innate immunity seems to be predominated in active severe disease state [5]. Innate immune cells are able to respond rapidly to skin injury and infection and secrete large amounts of inflammatory mediators. Besides Th17 cells, multiple innate immune cells could produce IL-17, including *γδ* T cells, natural killer (NK) cells, invariant natural killer (iNKT) cells, mast cells, macrophages, neutrophils, and IL-17 innate lymphoid cells. These innate-derived IL-17 act as a critical element in the immune response to self antigens and sustaining inflammation, involving in psoriasis pathogenesis [6]. Moreover, upon activation, the innate immune system could undergo epigenetic and metabolic reprogramming, leading to enhanced responsiveness to a second challenge [7]. This process is called “trained immunity,” in the nature of innate immune memory, which could contribute to detrimental outcomes in chronic inflammatory diseases [7]. Therefore, further investigation of innate immune status in psoriatic plaque is important to understand the pathogenesis of psoriasis and improve the therapeutic efficacy.

In this work, we applied the CIBERSORT algorithm with microarray data downloaded from the Gene Expression Omnibus (GEO) database, to calculate innate immune cell proportion in psoriatic skin. A list of related core genes was screened by correlation analysis with clinical characteristics. The combination study of innate immune cells and related genes supplies a better understanding of the innate immune response in psoriasis and provides clues for clinical application.

## 2. Materials and Methods

### 2.1. Microarray Data Acquisition and Preprocessing

Gene expression datasets and sample information were acquired from the GEO database (http://www.ncbi.nlm.nih.gov/geo). Eleven datasets (Affymetrix GPL570 Platform, Affymetrix Human Genome U133 Plus 2.0 Array) were downloaded, including GSE14905, GSE13355, GSE67853, GSE69967, GSE30999, GSE117468, GSE53552, GSE50790, GSE47751, GSE41663, and GSE78097. Samples acquired prior to therapy with brodalumab or ustekinumab were enrolled in this study. In total, skin samples from 91 healthy donors, paired nonlesional and lesional samples from 363 psoriasis patients, and lesional samples from additional 42 patients (the paired nonlesional skin were unavailable) were selected for further analysis. Data preprocessing included background correction and quantile normalization by the Affy package [8] in R (v3.6.3). Then, probes were converted into gene symbols. After batch normalization by the sva package [9], the merged data was processed to remove null values by the KNN method in the impute package [10].

### 2.2. Immune Infiltration Analysis

CIBERSORT [11] is an analytical tool to estimate the proportion of diversified cell types from complex tissues based on gene expression data. The gene signature file LM22 was used to identify immune cells infiltrated in the skin. Samples with CIBERSORT *p* value < 0.05 were selected for further analysis.

### 2.3. Identification of Differentially Expressed Innate Immune Genes

The limma package [12] was utilized to screen the differentially expressed genes (DEGs) between psoriasis lesional and control (nonlesional or normal) skin. The filter criteria of DEGs were set as adjusted *p* value < 0.05 and log2 | fold change (FC) | >1. The list of genes involved in the innate immune modulation was downloaded from the http://www.innatedb.com database [13]. The innate immune genes among the DEGs were identified by the Venn diagram.

### 2.4. GSEA-Based KEGG Pathway Analysis

Gene set enrichment analysis (GSEA) (http://software.broadinstitute.org/gsea/index.jsp) is an analytical method to identify if predefined gene sets are functionally enriched, by comparing genes from different groups [14]. According to the median value of the core gene, samples were divided into the high-expression group and the low-expression group. Kyoto Encyclopedia of Genes and Genomes (KEGG) pathway enrichment analysis was carried out by GSEA. The nominal *p* value < 0.05 and false discovery rate < 25% were set as the cut-off criteria.

### 2.5. Statistical Analysis

All statistical analyses were conducted in R v3.6.3 software. The proportion of innate immune cells between different groups was compared by the Wilcoxon rank-sum test. Spearman correlation analysis was performed to reveal the relationship between the expression of genes and parameters, such as disease severity, treatment response, and innate immune cells. *p* value < 0.05 was considered statistically significant.

## 3. Results

### 3.1. Composition of Innate Immune Cells in Psoriatic Lesion

**Figure 1:**
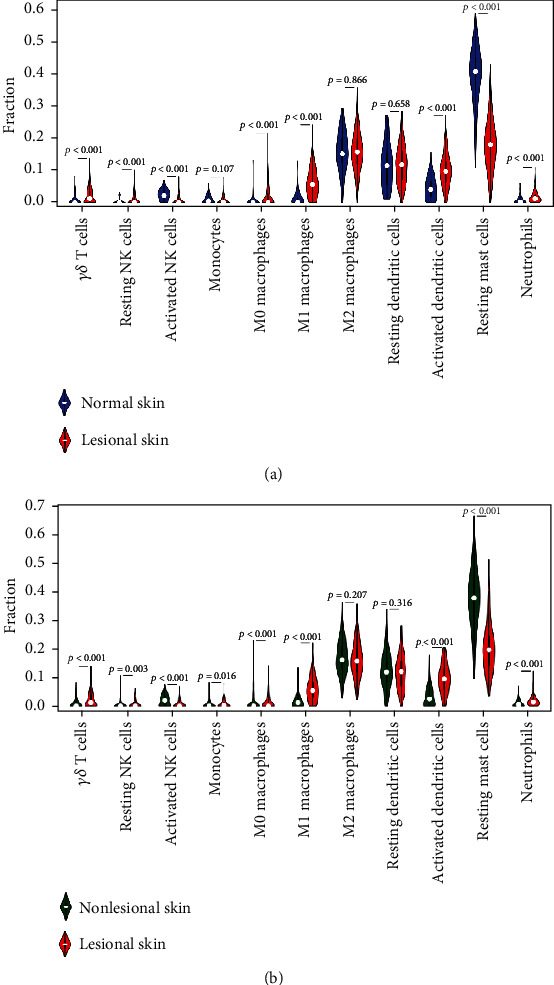
Landscape of innate immune cell infiltration in psoriasis. (a) Comparison of innate immune cells between psoriasis lesional skin (*N* = 316) and normal skin (*N* = 64). (b) Comparison of innate immune cells between psoriasis lesional skin (*N* = 182) and nonlesional skin (*N* = 182). *p* < 0.05 was considered statistically significant. *N*: number.

The fraction of infiltrated innate immune cells was evaluated in psoriasis lesional, nonlesional, and normal skin. Samples with CIBERSORT *p* < 0.05 were eligible for further analysis, including normal skin from 64 healthy donors and lesional skin from 316 patients, 182 of whom had matched nonlesional samples. The expression signature of 11 innate immune cell types was analyzed. Activated mast cells and eosinophils were excluded, as they were not significantly found in these samples after calculation. As shown in Figure 1, *γδ* T cells, resting NK cells, M0 macrophages, M1 macrophages, activated dendritic cells, and neutrophils were upregulated in psoriasis lesional skin, compared with nonlesional or normal skin. Activated NK cells and resting mast cells were downregulated.

### 3.2. Association between Innate Immune Cells and Clinical Manifestation in Psoriasis Patients

**Figure 2:**
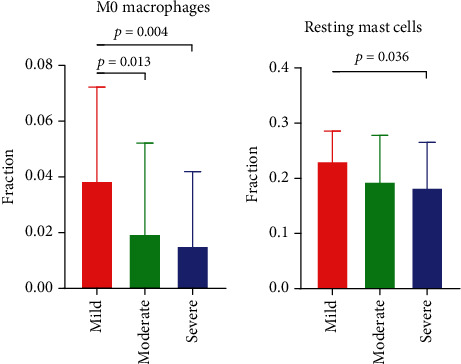
The differences of innate immune cell infiltration among mild, moderate, and severe psoriasis. The fraction of M0 macrophages or resting mast cells was significantly different.

In this study, the severity of psoriasis was indicated by Psoriasis Area and Severity Index (PASI). Based on PASI, the severity was classified into three levels: mild (0-12), moderate (12-18), and severe (>18) (Table 1). Among these innate immune cells, the proportion of M0 macrophages and resting mast cells showed correlation with disease severity (Figure 2). The fraction of M0 macrophages was lower in moderate or severe psoriasis, compared with mild psoriasis. The proportion of resting mast cells in severe was also lower than in mild psoriasis. The therapeutic efficacy was represented by the drug's PASI efficacy profile, which is the percentage of reduction in the PASI score after 12 weeks of treatment (Table 1). There was no correlation between innate immune cell fraction and therapeutic efficacy of brodalumab or ustekinumab (data not shown).

### 3.3. Identification of Differentially Expressed Innate Immune Genes

**Figure 3:**
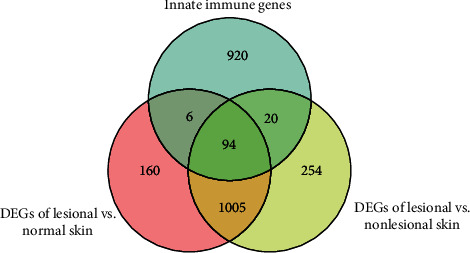
Venn plot illustrating differentially expressed innate immune genes.

After standardization of the microarray data, 1265 DEGs involved in psoriatic lesion were screened, compared with normal skin. Meanwhile, there were 1373 DEGs between the lesional skin and the nonlesional skin. 1040 genes involved in the innate immune response were downloaded from the InnateDB database. The Venn plot identified 94 common genes among these datasets (Figure 3).

### 3.4. Discovery of Core Genes Associated with Clinical Parameters

**Figure 4:**
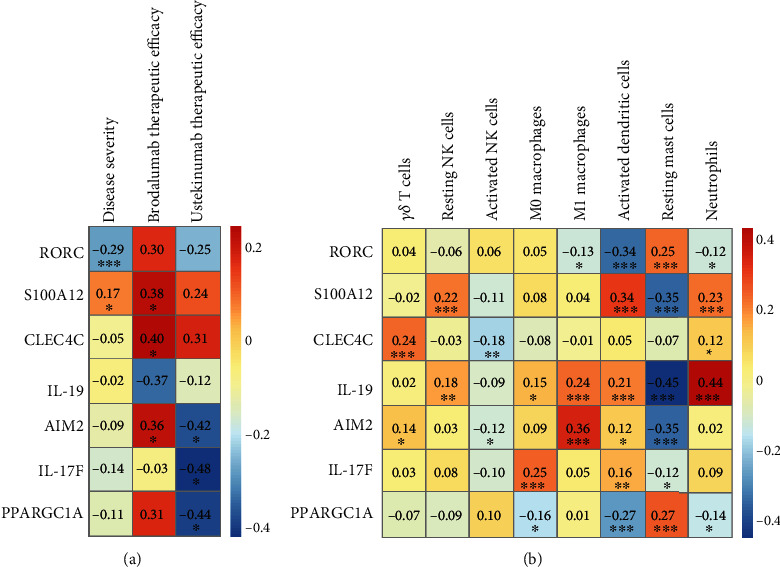
Correlation of core genes with clinical features and innate immune cells. (a) Spearman correlation analysis between core genes and clinical characteristics, including disease severity and therapeutic efficacy of brodalumab and ustekinumab. (b) Spearman correlation analysis of core genes and proportion of innate immune cells. ^∗^*p* < 0.05, ^∗∗^*p* < 0.01, and ^∗∗∗^*p* < 0.001.

Figure 4(a) shows that 7 differentially expressed innate immune genes were significantly correlated with clinical indicators. These genes were identified as core genes. Among these genes, RORC and S100A12 were related to disease severity. S100A12, CLEC4C, and AIM2 were positively correlated with the therapeutic efficacy of brodalumab, while IL-19 was negatively correlated. AIM2, IL-17F, and PPARGC1A were negatively associated with ustekinumab treatment response.

### 3.5. Comprehensive Correlation Analysis of Core Genes and Innate Immune Cells

As shown in Figure 4(b), core genes showed close connection with the fraction of innate immune cells. Genes such as S100A12, IL-19, AIM2, and IL-17F were positively correlated with activated dendritic cells and negatively correlated with resting mast cells, while RORC and PPARGC1A were complete opposite. IL-19 and IL-17F were positively related to M0 macrophages; meanwhile, IL-19 and AIM2 were positively associated with M1 macrophages.

### 3.6. Predicted Function of Core Genes in Psoriasis

**Figure 5:**
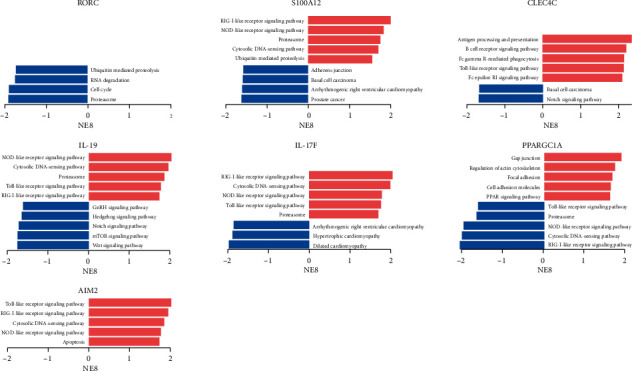
Pathways associated with core gene alteration predicted by GSEA. The alteration was the comparison between the high-expression group and the low-expression group. Pathways enriched in phenotype high were shown in red (right), and pathways enriched in phenotype low were shown in blue (left). All gene sets were significantly enriched at nominal *p* < 5% and false discovery rate < 25%.

To explore the potential function of core genes in psoriasis, GSEA was performed for KEGG analysis (Figure 5). All or part of pathways involving RIG-I-like receptor signaling, NOD-like receptor signaling, toll-like receptor signaling, and cytosolic DNA-sensing pathway were significantly enriched and positively correlated with S100A12, CLEC4C, IL-19, IL-17F, and AIM2, whereas negatively correlated with PPARGC1A. The pathways associated with the ubiquitination-proteasome system were significantly regulated by RORC and S100A12 alteration in psoriasis.

## 4. Discussion

Psoriasis is an immune-mediated skin disease. Although great advances have been made in search for psoriasis pathogenesis and treatment, therapeutic approaches are still limited for the cure. In this work, we took advantage of big data analysis, in combination with CIBERSORT algorithms, to explore novel insights into the innate immune status in psoriasis and identify prognostic biomarkers.

We found considerable differences of innate immune cell composition between psoriatic skin and the control group (nonlesional or normal skin). Results revealed that *γδ* T cells, resting NK cells, M0 macrophages, M1 macrophages, activated dendritic cells, and neutrophils were significantly increased in psoriatic skin, while resting mast cells and active NK cells were significantly decreased. Moreover, the proportion of M0 macrophages was associated with disease severity and treatment efficacy. Macrophages are considered to originate from peripheral monocytes, which enter tissue and differentiate under stimulation of the local environment. Unactivated M0 macrophages could polarize into M1 or M2 macrophages under different cytokines. M1 macrophages are implicated in activating inflammatory response, while M2 macrophages are involved in the resolution of inflammation [15]. A previous study showed that when peripheral monocytes derived from psoriasis patients were induced in vitro, the ratio of M1/M2 macrophage polarization was higher, compared with healthy control [16]. It indicates that monocytes in psoriasis might undergo a reprogramming process-trained immunity [7], acquiring the preference to M1 polarization. Derived from monocytes, M0 macrophages in psoriatic plaque might also be reprogrammed in the same way. In addition, IL-23 promoted the expression of IL-17A, IL-17F, IFN-*γ*, and IL-22 in M0 macrophages, and IL-23-treated M0 macrophages significantly enhanced psoriasis-like dermatitis in the mouse model [17]. Moreover, M0 macrophages were susceptible to this IL-23-driven M (IL-23) differentiation, while surprisingly, M1 and M2 macrophages were highly resistant to polarization into M (IL-23) macrophages [17]. We unexpectedly found that IL-17F expression was positively related to the proportion of M0 macrophages in lesional skin. IL-23 is highly expressed in psoriatic skin. Under IL-23 stimulation, M0 macrophages might play an unexpected and important effect in psoriasis pathogenesis.

*γδ* T cells were the source of IL-17 and increased in psoriatic skin [18]. Murine models lacking *γδ* T revealed inhibited psoriasiform symptoms and inflammation [18, 19]. In psoriasis vulgaris patient blood, NK cells were reported to have reduced cytotoxicity and production of proinflammatory cytokines TNF-*α* and IFN-*γ* [20]. However, another study showed that NK cells isolated from psoriatic skin secreted more IFN-*γ* [21]. Researches about NK cells involved in psoriasis are insufficient, and the role in pathogenesis is not clear. Activated DCs played a pivotal role in psoriasis pathogenesis. They not only initiated the inflammation but also sustained it [22]. Activated mast cells were elevated in psoriatic plaque and produced IL-17 and IL-22 [23]. In our analysis, the fraction of activated mast cells was rare. It is possible that CIBERSORT enumerates mainly IgE-activated mast cells, for which gene expression signature employed for deconvolution was acquired from mast cells induced by IgE [11], while activation of mast cells by IL-1 was an important process in skin inflammation and implicated in psoriasis pathogenesis [24]. Recent studies revealed that neutrophils in psoriatic plaque generated abundant neutrophil extracellular trap, degranulation, and respiratory burst with ROS production, contributing to the pathogenesis of psoriasis [25]. Collectively, different innate immune cells infiltrated in the skin could play various roles in psoriasis development and progression. The specific function of some types remains to be elucidated.

Further analysis found that RORC and S100A12 were associated with psoriasis severity. The nuclear receptor retinoic acid receptor-related orphan receptor gamma (murine form “ROR*γ*” or human form “RORC”) was a key transcription factor for the production of IL-17 and involved in the inflammatory response of psoriasis [26]. Compared with severe psoriasis, mild psoriasis was characterized by higher IL-17A expression in psoriatic lesion [27], which is consistent with the alteration of RORC in our study. Among S100 proteins, the serum S100A12 level revealed the closest association with psoriasis disease activity-PASI [28]. We also found this positive correlation in psoriatic plaque. Ubiquitination, a type of posttranslational modification, is viewed as an important regulator in psoriasis pathogenesis, through modulation of key transcription factors or signaling molecules [29]. ROR*γ*t, a splice variant of ROR*γ*, could be modulated by both ubiquitination and deubiquitylation [29]. In this work, GSEA analysis indicated that low RORC expression or high S100A12 expression was associated with the ubiquitination-proteasome system. This suggests that the expression of RORC and S100A12 might be regulated by ubiquitination, which is related to the modulation of psoriasis severity.

Ustekinumab and brodalumab are monoclonal antibodies applied for psoriasis treatment. Ustekinumab can inhibit the IL-23 pathway by binding to the common p40 subunit of IL-12/IL-23, while brodalumab targets IL-17 receptor A and blocks the IL-17 pathway. This study revealed that multiple genes were related to therapeutic efficacy. After activation, AIM2 assembles inflammasome, driving IL-1*β* secretion and contributing to psoriasis [30]. Ngoungoure and Owona [31] reported that M1-polarized macrophages expressed higher AIM2, compared with resting THP-1 macrophages. In this work, AIM2 expression was positively correlated with M1 macrophage fraction in psoriatic plaque, indicating that AIM2 might regulate psoriasis progression through modulating M1 macrophages. However, its relationship with treatment response was inconsistent in brodalumab and ustekinumab. AIM2 might play a different effect on the IL-17 and IL-23 pathway, and it needs to be further explored.

CLEC4C is a plasmacytoid dendritic cell- (pDC-) specific receptor. Cross-linking CLEC4C with monoclonal antibodies could lead to receptor internalization, rapid Ca^2+^ influx, and inhibition of IFN-I response in pDCs [32]. We found CLEC4C expression was positively correlated with therapeutic efficacy with brodalumab, which might be a result of CLEC4C engagement induced by monoclonal antibody brodalumab. CLEC4C could not only predict brodalumab treatment response but also be exploited as a target for selecting therapeutic monoclonal antibodies. IL-19 could amplify IL-17A effects on keratinocytes in psoriasis [33]. We found IL-19 expression was negatively correlated with anti-IL-17RA therapy.

IL-17F is a subtype of IL-17. A previous study reported that IL-17F polymorphism rs763780 was associated with better efficacy of anti-TNF therapy [34]. In this study, low IL-17F expression was also linked to better response to anti-IL-23 treatment. PPARGC1A interacts with PPAR*γ* and is a transcriptional coactivator. During acute colitis, PPARGC1A activation in intestinal CD11b-CD103+DCs promoted the production of retinoic acid, which subsequently acted on CD11b+CD103+DCs to suppress IL-23 secretion [35]. There is no report about PPARGC1A in psoriasis. Based on previous work and our finding of negative correlation between PPARGC1A and activated DCs, we presume that PPARGC1A might inhibit DC production of IL-23. Therapy by blocking IL-23 might not be effective in moderate-to-severe psoriasis patients with high PPARGC1A expression. In sum, these results indicate that these genes may have important clinical implications in psoriasis and deserve deeper investigation.

However, this study has limitation. The number of samples for clinical association analysis was relatively small. Large sample clinical studies need to be conducted to validate our results.

## 5. Conclusions

Our study provides new clues about innate immune status in psoriatic skin. Eight types of innate immune cells showed obvious association with psoriasis, and M0 macrophage or resting mast cell proportion revealed significant differences when comparing disease severity. Seven core genes related to clinical parameters were analyzed and may play crucial roles in disease progression. Further study of these genes in psoriasis could identify targets for therapy and biomarkers for individualizing treatment strategies.

## Figures and Tables

**Table 1:** Clinical characteristics of patients with psoriasis.

Clinical characteristics	Values	Cases
Disease severity	Mild	13
	Moderate	59
	Severe	60
Brodalumab therapeutic efficacy (%)	89.94 ± 21.05	32
Ustekinumab therapeutic efficacy (%)	79.82 ± 17.66	26

## Data Availability

The datasets analyzed in this study are openly available in the GEO database at http://www.ncbi.nlm.nih.gov/geo.
